# Linking Protein Stability to Pathogenicity: Predicting Clinical Significance of Single-Missense Mutations in Ocular Proteins Using Machine Learning

**DOI:** 10.3390/ijms252111649

**Published:** 2024-10-30

**Authors:** Iyad Majid, Yuri V. Sergeev

**Affiliations:** Ophthalmic Genetics and Visual Function Branch, National Eye Institute, National Institute of Health, Bethesda, MD 20892, USA

**Keywords:** inherited eye disease, genetic mutations, protein stability, pathogenicity prediction, machine learning, computational biology

## Abstract

Understanding the effect of single-missense mutations on protein stability is crucial for clinical decision-making and therapeutic development. The impact of these mutations on protein stability and 3D structure remains underexplored. Here, we developed a program to investigate the relationship between pathogenic mutations with protein unfolding and compared seven machine learning (ML) models to predict the clinical significance of single-missense mutations with unknown impacts, based on protein stability parameters. We analyzed seven proteins associated with ocular disease-causing genes. The program revealed an R-squared value of 0.846 using Decision Tree Regression between pathogenic mutations and decreased protein stability, with 96.20% of pathogenic mutations in RPE65 leading to protein instability. Among the ML models, Random Forest achieved the highest AUC (0.922) and PR AUC (0.879) in predicting the clinical significance of mutations with unknown effects. Our findings indicate that most pathogenic mutations affecting protein stability occur in alpha-helices, beta-pleated sheets, and active sites. This study suggests that protein stability can serve as a valuable parameter for interpreting the clinical significance of single-missense mutations in ocular proteins.

## 1. Introduction

The primary structure of a protein dictates its three-dimensional shape and biological function in the human body. Genetic mutations play a critical role in impacting the clinical significance of various diseases by directly altering the structure of a protein [1,2]. Mutations may also disrupt the secondary and tertiary structures; a protein’s secondary structure is stabilized by hydrogen bonds between the backbones of peptides while the tertiary structure is determined by a variety of interactions between the side-chain residues of amino acids [3]. A protein’s overall three-dimensional shape is identified as the most thermodynamically stable confirmation, and thus proteins are typically stable in physiological conditions [3].

Understanding the impact of genetic mutations on protein structure and stability is critical for predicting their clinical significance—whether a mutation is pathogenic or benign. Mutations may destabilize a protein’s tertiary structure, leading to either a partial or complete loss of function or a gain in toxicity [4,5,6], both of which are common mechanisms underlying many genetic disorders [1,3,7,8]. Thus, predicting the pathogenicity or benign nature of mutations based on their effects on protein stability is essential for understanding inherited disease mechanisms and developing therapeutic strategies. Our team previously developed an algorithm called the Unfolding Mutation Screen (UMS) that calculates an empirical estimate of a genetic mutation’s ability to cause protein misfolding using the predicted unfolding fractions of protein residues, which are derived from the free energy changes between the mutant and wild-type protein structures [8,9]. The results of this work were used for the evaluation of the mutation effect on protein stability. These parameters were previously pre-calculated for more than 100 proteins from inherited eye disease and are currently available from the ocular proteome website of NEI Data Commons. These micro-level parameters provide a more direct measure of how mutations impact protein stability.

Recent advances in computational biology, particularly machine learning (ML) models, have enabled the clinical significance of protein residue mutations using large-scale data analysis. EVE (Evolutionary Variant Effect) is a deep-learning model that predicts if certain mutations are pathogenic or benign by analyzing evolutionary sequence data [10]. This macro-level approach offers insights into the broader physiological effects of missense mutations and their pathogenicity, but does not directly account for the physical, structural changes that can affect stability.

Despite recent computational advances, there still remains a clear link between disruptions in a protein’s three-dimensional stability and its functional consequences at the level of inherited disease, such as whether a mutation affecting stability leads to a pathogenic significance or is benign. The purpose of this work is to address the relationship between the clinical significance of single-missense mutations and protein stability. Additionally, we compare and validate various ML models to predict the pathogenicity of single-missense mutations with unknown clinical significance, using protein unfolding fractions calculated by UMS. We hypothesize that protein stability parameters, derived from UMS, can serve as a valid predictor of the clinical impact of these mutations.

Unlike previous studies that primarily rely on sequence-based or evolutionary data [11,12,13], our method leverages data related to protein folding and stability, providing a more direct measure of the structural impacts of mutations. This approach offers a new perspective on the genotype–phenotype relationship, particularly in the context of ocular proteins, and aims to enhance the accuracy of pathogenicity predictions for mutations with unknown clinical significance.

To predict the pathogenicity of mutations with unknown clinical significance, we employed four ML algorithms: Decision Tree, Random Forest, Extreme Gradient Boosting (XGBoost), and Support Vector Machine (SVM) (assessed on 10 July 2024). These algorithms are powerful tools for capturing complex patterns in the data and have been widely used in genotype-to-phenotype relationship predictions [14,15]. We also explored the performance of three neural networks: a Dense model, an LSTM model, and an Ensemble network. Neural networks are particularly well-suited for modeling non-linear relationships and handling large datasets, making them beneficial for predicting the effects of genetic mutations on protein stability and function. By comparing the performance of these ML models and neural networks, we aim to identify the most effective approach for predicting genetic mutation outcomes based on protein stability.

Here, we present a novel program that links pathogenic mutations to protein stability and predicts the clinical significance of single-missense mutations with effects based on stability parameters across seven ML models. Our program was applied to seven proteins associated with inherited ocular diseases, including hemoglobin subunit beta (HBB), tyrosinase-related protein 1 (TYRP1), rhodopsin (RHO), retinal pigment epithelium-specific 65 kDa protein (RPE65), dehydrodolichyl diphosphate synthase (DHDDS), inosine-5′-monophosphate dehydrogenase 1 (IMPDH1), and kelch-like protein 7 (KLHL7). The focus on these proteins was driven by the availability of well-curated data and the relevance to inherited eye disorders, which aligns with the scope of our study. To our knowledge, this study is the first to predict the effects of mutations with unknown clinical significance based on protein stability parameters, offering new insights into the genotype–phenotype relationships. Our project represents a proof-of-concept, demonstrating the predictive power of protein stability parameters in predicting the pathogenicity of single-missense mutations with unknown clinical significance.

## 2. Results

### 2.1. Average Unfolding Fraction and Average Pathogenicity Score

As a preliminary study, we developed a program to compare average pathogenicity and average unfolding fractions quantitatively and qualitatively, or clinical significance and protein stability factors, for each amino acid residue in seven ocular proteins. Table 1 shows the number of pathogenic mutations listed in ClinVar that overlap, with high average pathogenicity scores and high unfolding fractions. The percentages indicate how often these two factors coincide, highlighting the genes with the highest overlap. Of the seven proteins analyzed, KLHL7 was made up of the greatest number of amino acid residues; however, RHO contained the greatest number of pathogenic mutations listed in ClinVar. RPE65 had the highest percentage of overlaps between high average-pathogenicity scores and unfolding fractions (96.20%), followed by KLHL7 (94.40%) and RHO (91.40%).

**Figure 1:**
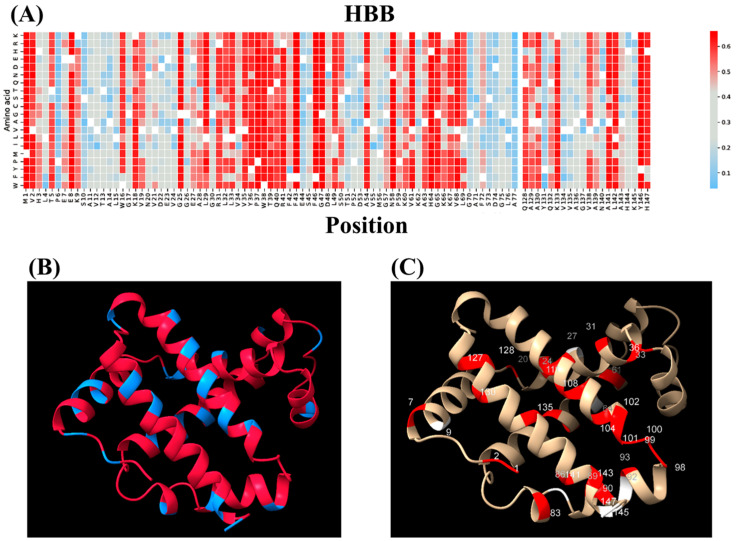
(**A**) Heatmap of EVE pathogenicity scores of HBB. For each amino acid, the scores were summed up and divided by 19. (**B**) Average unfolding fractions are represented on HBB, where high and low unfolding fractions are highlighted in red and blue, respectively. These values were pulled from UMS. (**C**) Overlap of pathogenic mutations and their impact on protein unfolding, where high pathogenicity score and unfolding fraction matches are highlighted in red, and matched residues not listed as pathogenic in ClinVar are highlighted in white.

The general idea of finding the relationship between protein stability and the clinical significance of single-missense mutations is illustrated in Figure 1, in the example of protein HBB. Panels A–C of Figure 1 provide a heatmap of EVE pathogenicity scores for HBB, along with the qualitative analysis of average unfolding fractions and high average-pathogenicity scores and unfolding fractions overlaps depicted on the protein structure. The procedure, which was applied for the HBB protein. was applied to other protein structures listed in Materials and Methods. To calculate the R-squared value for the data, we utilized Decision Tree Regression, a non-linear model that measures how well the predictions from the regression model approximate the real data points. The regression model calculated an R-squared value of 0.846 between pathogenic mutations and protein stability parameters. The pathogenicity and unfolding fraction scores for each mutation of all seven proteins investigated are provided in Table S1 in the Supplementary Materials.

### 2.2. Performance of Machine Learning Algorithms on Pathogenicity Prediction

We used four frequently used ML algorithms (Decision Tree, Random Forest, XGBoost, and SVM) to predict the pathogenicity of mutations with unknown clinical significance in ClinVar. Our criteria for a valid model prioritize an area-under-the-curve (AUC) score greater than 0.800 and a precision-recall AUC (PR AUC) score greater than 0.850. The six performance metrics for the four ML algorithms are provided in Table 2.

**Figure 2:**
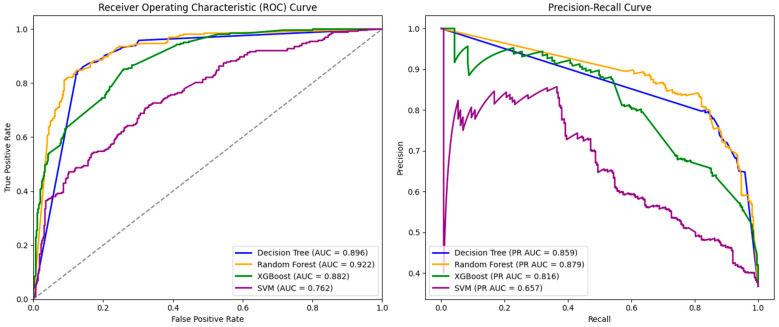
Comparison of areas determined under the Receiver Operating Characteristic Curve illustrating the performance of a binary classifier model and the Precision–Recall Curve used for evaluating the performance of binary classification algorithms. The areas are characterized by the AUC (**left**) and PR AUC (**right**) curves of all four ML algorithms.

The Random Forest algorithm achieved the highest AUC (0.922), Recall (0.878), and PR AUC (0.847) scores. The Decision Tree algorithm achieved the greatest F1-score (0.813), accuracy (0.852), and precision (0.759). Of note, the SVM algorithm achieved the lowest results across all six metrics. In addition, a plot of the AUC and PR AUC curves for each algorithm is provided in Figure 2. The Random Forest algorithm achieved the highest AUC (0.922), followed by Decision Tree (0.896), XGBoost (0.882), and SVM (0.762). Of note, Random Forest performed significantly better than the latter two models (*p* < 0.005), but not significantly better than the Decision Tree (*p* = 0.414); in addition, XGBoost and Decision Tree performed significantly better than SVM (*p* < 0.005). The Random Forest algorithm also achieved the greatest PR AUC (0.879), followed by Decision Tree (0.859), Decision Tree (0.816), and SVM (0.657). The Decision Tree algorithm achieved the highest accuracy (0.852), followed by Random Forest (0.849), XGBoost (0.782), and SVM (0.690). Regarding precision, Decision Tree achieved the highest score (0.759), while SVM achieved the lowest score (0.569). Regarding recall, Random Forest achieved the highest score (0.878) and SVM achieved the lowest result (0.643). We conclude that the Random Forest algorithm performs the best in predicting the pathogenicity of mutations with unknown clinical significance.

### 2.3. Performance of Neural Networks on Pathogenicity Prediction

**Figure 3:**
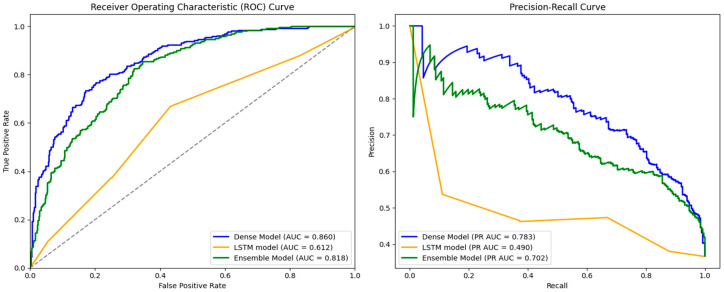
Comparison of areas determined under the Receiver Operating Characteristic Curve and the Precision–Recall Curve. AUC (**left**) and PR AUC (**right**) curves of all three neural networks.

We also developed three neural networks (Dense, LSTM, and Ensemble models) to predict the pathogenicity of mutations with unknown clinical significance in ClinVar, and compared their performances to the ML algorithms. Table 3 lists the performance of the models across all six metrics. The Dense model achieved the highest AUC (0.860), F1-score (0.708), accuracy (0.779), precision (0.712), and PR AUC (0.783) scores. Of note, the LST model achieved the lowest results across all metrics except recall (1.000). Figure 3 provides a plot of the AUC and PR AUC curves for the three neural networks.

The Dense model achieved the highest AUC and PR AUC (0.860 and 0.783, respectively), followed by the Ensemble network (0.818, 0.702) and the LSTM model (0.612, 0.490).

As stated above, we prioritize AUC (a score greater than 0.800) and PR AUC (0.850) when determining if a model’s performance is valid. The Dense model achieved the highest AUC among the models (0.860, *p* < 0.005), followed by the Ensemble network (0.818) and the LSTM model (0.612). Additionally, the Dense model achieved the highest F1-score, accuracy, precision, and PR AUC (0.708, 0.779, 0.712, and 0.783, respectively), compared to the Ensemble network (0.694, 0.751, 0.584, and 0.702) and the LSTM model (0.537, 0.367, 0.367, and 0.490). Of note, the LSTM model achieved the highest recall score (1.000), outperforming both the Ensemble (0.856) and Dense (0.703) models. We conclude that, of the neural networks, the Dense model performs the best at predicting the pathogenicity of mutations with unknown clinical significance.

In predicting the pathogenicity of mutations with unknown clinical significance, the ML algorithms outperformed the neural networks. Regarding AUC and PR AUC, the Random Forest algorithm achieved the highest scores (0.922 and 0.879, respectively), followed by Decision Tree (0.896, 0.859), XGBoost (0.882, 0.816), and the Dense model (0.860, 0.783). The Decision Tree algorithm achieved the highest F1-score (0.813), followed by Random Forest (0.811), XGBoost (0.721), and the Dense model (0.708). However, in terms of recall the LSTM model achieved the highest performance (1.000), followed by Random Forest (0.878) and Decision tree (0.875). Our findings suggest that linear regression ML algorithms perform better at predicting the clinical significance of unknown mutations from protein stability parameters, compared to neural networks.

### 2.4. Structural Validation of Predictions

**Figure 4:**
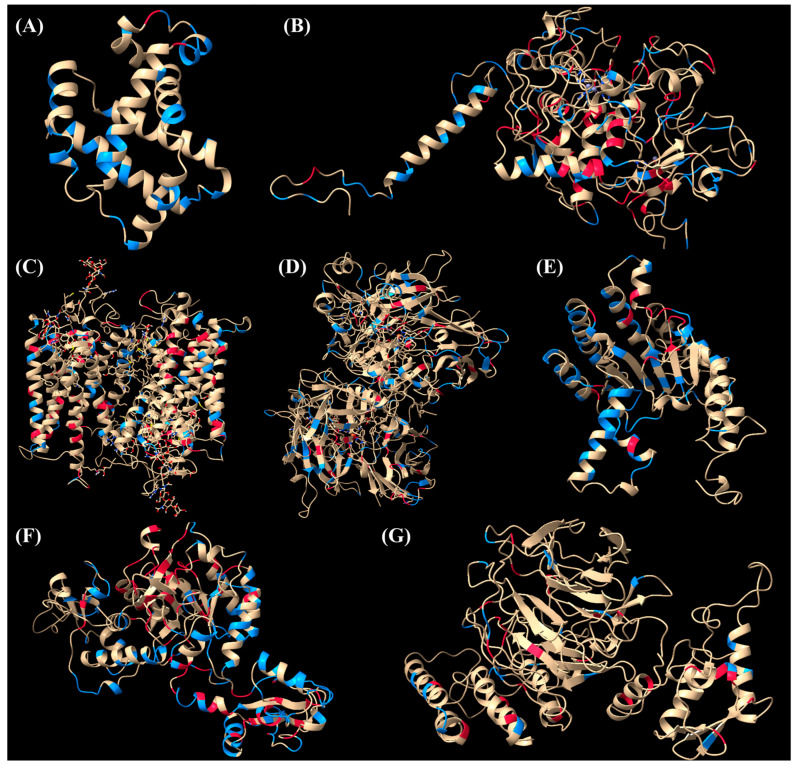
Predictions of the clinical significance of mutations with unknown significance by Random Forest. Red and blue residues are predicted to be pathogenic and benign, respectively. The proteins are as follows: (**A**) HBB, (**B**) TYRP1, (**C**) RHO, (**D**) RPE65, (**E**) DHDDS, (**F**) IMPDH1, (**G**) KLHL7.

To validate the predictions of the Random Forest algorithm, the 716 mutated residues with unknown clinical significance were visualized in ChimeraX. The Random Forest algorithm achieved an accuracy of 0.880 and an F1-score of 0.810 when predicting pathogenic cases, and an accuracy of 0.830 and an F1-score of 0.870 when predicting benign cases. The predicted mutations are highlighted in red and blue for pathogenic or benign, respectively, in Figure 4.

For example, in HBB, mutations V134L and D74H were predicted as benign, while N58S and G47R were predicted as pathogenic. For TYRP1, D308A was predicted as benign, while P346R and R87Q were predicted as pathogenic. In RHO, E181Q was predicted as pathogenic. These predictions were corroborated by the existing biochemical literature, which supports the validity of our model’s predictions and the role of structural context in determining mutation pathogenicity. The unfolding fraction and location of mutations predicted to be pathogenic are provided in Table S2 in the Supplementary Materials. Of note, most pathogenic mutations occurred in alpha helixes, beta pleated sheets, and regions near the active site, indicating an effect on the protein’s secondary structure and overall stability.

## 3. Discussion

Despite extensive research, the impact of single-missense mutations with unknown clinical significance on protein stability and structure remains unclear. Our study addresses this gap by investigating the relationship between inherited mutations causing protein misfolding and pathogenic clinical significance using a novel program and evaluating the predictive performance of seven ML models. We focused on seven ocular proteins associated with inherited eye disease (HBB, TYRP1, RHO, RPE65, DHDDS, IMPDH1, and KLHL7). Using Decision Tree Regression, we found a strong association (R-square of 0.846) between pathogenic mutations and reduced protein stability, particularly noting that 96.20% of pathogenic mutations in RPE65 lead to protein instability. Among the models tested, Random Forest performed the best in predicting the clinical significance of mutations with unknown impacts. Our findings suggest that protein stability is a key parameter for interpreting the clinical significance of these mutations, particularly in key structural elements like alpha-helices, beta-sheets, and regions near active sites.

In our comparison of ML algorithms and neural networks, the machine learning models greatly outperformed the neural networks in predicting the pathogenicity of mutations with unknown clinical significance. Of the ML algorithms, Random Forest achieved the highest performance, with an AUC of 0.922 and a PR AUC of 0.879; among the deep learning algorithms, the Dense model achieved the highest performance, with an AUC of 0.860 and a PR AUC of 0.783. Of note, the LSTM model achieved a perfect recall (1.000), the highest compared to all other models, but performed the poorest compared to all models evaluated regarding AUC and PR AUC. The differences in performance between the ML models underscore the unique advantages and limitations of each type of model.

Traditional ML algorithms, such as Random Forest and XGBoost, excelled due to their ability to capture complex patterns in the data through ensemble learning techniques by combining the outputs of multiple decision trees to improve accuracy and generalization while reducing overfitting [12,13]. The poor performance of SVM can be attributed to its sensitivity to hyperparameter tuning and its tendency to struggle with high-dimensional features, such as our several-hundred amino acid pathogenicity and stability parameters, as well as non-linear relationships in the data. The neural networks, including the Dense and LSTM models, likely underperformed due to the limited dataset size, which may have been insufficient for proper generalization. The small dataset, combined with the model’s complex architectures, also increased the risk of overfitting [16]. Additionally, the structured protein stability data used in this study are better suited to traditional models like Random Forest, whereas neural networks typically excel with unstructured or sequential data, such as images. A solution to a low quantity of data is data augmentation, which is commonly used to improve model performance [17], but was not applicable in this study as altering the input data (protein stability parameters) would result in fundamentally different protein characteristics.

Our analysis of residues with high average-unfolding fractions and pathogenicity revealed that pathogenic mutations predominantly occur in alpha helices and beta-pleated sheets, structures critical for protein function. Mutations near active sites were also highlighted, further emphasizing the potential of these mutations to disrupt catalytic activity. This observation supports the broader understanding that the integrity of these secondary structures is crucial for protein function, and mutations that disrupt them can lead to misfolding, loss of stability, and impaired function. Using ChimeraX-1.8, we validated the predictions made by the Random Forest algorithm for several mutations with unknown clinical significance, comparing them with the existing literature and biochemical data. For example, in HBB, the algorithm predicted the mutations V134L and D74H to be benign, and N58S and G47R to be pathogenic. Position 74 of HBB is on the outside of the protein and does not interact with the nearby hemoglobin subunit alpha [18,19], which forms the hemoglobin tetramer necessary for blood cells to transport blood oxygen [20]. In addition, the non-polar, neutral valine is substituted for the non-polar, neutral leucine, preserving the residue’s shape, size, charge, and reactivity. The D74H has been reported to improve hemoglobin binding affinity for oxygen [21]. Of interest, the mutation substitutes the acidic, polar aspartate for the basic, bulky, and polar histidine. No reported patients with the mutation V134L or D74H have been reported to have Beta Thalassemia [22,23]. The mutation N58S is reported to also increase binding affinity to oxygen, but is predicted to be unstable [19,24]. The mutation substitutes asparagine (a large, polar residue) for serine (a small, polar residue). The variant G47R may alter the physicochemical properties of the protein [25], as the neutral, non-polar glycine is replaced with a basic, polar arginine, and is found to cause increased dissociation between the hemoglobin alpha and beta subunits [25,26].

Other mutation predictions validated were P346R, R87Q, and D308A in TYRP1, and E181Q in RHO. P346R, predicted to be pathogenic by Random Forest, is found to have deleterious effects on protein function and structure via in silico analysis by GeneDx and Invitae [27]. In addition, the variant replaces the neutral, non-polar proline with a basic, polar arginine. R87Q, also predicted to be pathogenic, substitutes arginine (basic and polar) for glutamine (neutral and polar), and has been observed in patients presenting with TYRP1-related syndromes [28]. Our group previously found that residue 87 is found in the Cys-rich domain of TYRP1, and a substitution for glycine showed OCA3-related phenotypic changes [29]. Our group also found that a mutation at amino acid position 308 kept native protein conformation and did not affect enzymatic activity [29], as predicted by the model. However, we analyzed a substitution for alanine (a small, neutral, and non-polar residue) whereas our group previously analyzed a substitution for asparagine (a large, neutral, and polar residue). Regarding RHO, Random Forest predicted E181Q to be pathogenic. A patient was reported in the literature to have retinitis pigmentosa with a similar variant [30]. However, their mutation was to lysine (polar and basic), compared to the glutamine substitution analyzed (polar and neutral). E181 is present in extracellular loop 2 of rhodopsin and participates in hydrolysis of chromophore [31], so a mutation at that position is likely to cause a deleterious effect.

These findings align with the broader understanding that the integrity of secondary structures like alpha-helices and beta-sheets is crucial for protein function. Mutations that disrupt these secondary structures can lead to misfolding, loss of stability, and impaired function, common mechanisms underlying many genetic disorders [32,33,34,35,36,37]. Several studies indicate that residues with a higher density of contacts are more “resilient” to the deleterious effects of mutations [38,39]. In addition, the more stable a protein wild-type is, the less likely it will be affected by a mutation [40,41,42,43]. The proximity of these mutations to the active sites further emphasizes their potential to hinder the protein’s ability to interact with substrates or other molecules, thereby affecting its catalytic activity or binding affinity [44]. Moreover, proteins confined in tight environments or adsorbed onto surfaces can undergo significant structural alterations, including shifts in pKa values, which can substantially affect their stability and function, as highlighted in recent studies [45]. Therefore, it is essential to consider not just the mutation’s location within the protein sequence, but also its structural context when assessing its pathogenic potential.

Understanding how single-missense mutations affect protein stability is crucial for clinical decision-making. In essence, our work bridges the gap between genetic mutations and their impact on protein stability, offering valuable tools for assessing the clinical significance of single-missense mutations, especially those with unknown impacts. Our models are particularly beneficial in the context of rare or novel mutations where experimental data is limited or unavailable. By accurately predicting the effects of these mutations from available protein stability parameters, our models can aid in early diagnosis, inform treatment strategies, and guide the development of targeted therapies. Furthermore, the insights gained from our study contribute to a deeper understanding of the molecular mechanisms underlying genetic disorders, ultimately supporting more personalized and effective healthcare solutions.

This study had several limitations. The clinical significance of protein mutations was derived from the EVE model [10], which, while supported by multiplexed assays of variant effects (MAVEs) and deep mutational scans [46,47], occasionally disagrees with ClinVar classifications. Additionally, while our algorithm successfully identified regions where high average pathogenicity overlaps with high unfolding fractions, the exact relationship between these structural disruptions and the clinical phenotypes is not fully understood. At present, our study takes into account only the destabilizing effect of inherited missense mutations and does not include information on the specific protein, the nature of the mutation, and the cellular environment. Future work should integrate additional structural data, such as protein dynamics and interaction networks, as well as biophysical characteristics of amino acids, to gain a more comprehensive understanding of how these mutations affect protein function. Our model was trained on only seven proteins, primarily related to retinal diseases, limiting the generalizability of our findings. Moreover, we only analyzed proteins with single-missense mutations.

Future work should integrate additional structural data, such as protein dynamics and interaction networks, to gain a more comprehensive understanding of how these mutations affect protein function. Expanding the analysis to include other post-translational modifications and environmental factors could also provide deeper insights into the complex interplay between protein structure, function, and disease. Using other tools, such as MetaDome-1.0.1 [48], to label protein mutations as pathogenic or benign could further enhance our understanding of mutation impacts on protein stability, and MuToN [49], for inferring the protein–protein binding-affinity changes upon mutations, with the geometric transformer network. Moreover, we plan to integrate additional mutation types, such as multiple mutations, indels, nonsense, and frameshift mutations, to further improve the scope of our model. In addition, we aim to extend the model to include protein mutations associated with other ocular diseases, including glaucoma and diabetic retinopathy.

## 4. Materials and Methods

We analyzed single-missense mutations across seven inherited disease-related ocular proteins—HBB, TYRP1, RHO, RPE65, DHDDS, IMPDH1, and KLHL7. HBB and TYRP1 are related to beta thalassemia [50] and oculocutaneous albinism 3 (OCA3) [51], respectively; the other five proteins are linked to retinitis pigmentosa [52,53,54,55].

The preliminary phase of this project was to determine a relationship between protein stability (unfolding fractions) and clinical significance (pathogenicity scores). The second phase of this project was to assess if ML models could predict the pathogenicity of mutations with unknown clinical significance from unfolding fractions obtained from the ocular proteome website, available at NEI Data Commons (https://neidatacommons.nei.nih.gov/ocular-proteome (accessed on 10 July 2024)). Regarding the second task, we compared four ML algorithms and three neural networks.

### 4.1. Dataset

Our project utilized three datasets to predict the pathogenicity of protein single-missense mutations with unknown clinical significance, based on the mutated protein’s stability. We extracted pathogenicity scores from EVE [10] (https://evemodel.org/ (accessed on 10 July 2024)) to assess the likelihood that a specific mutation would result in disease. The EVE model produces a continuous score, from 0 (benign) to 1 (pathogenic), of the likelihood a certain amino acid variant will result in a disease.

We also employed unfolding fractions determined from protein atomic structures or homology models from the NEI Commons Proteome website (https://neicommons.nei.nih.gov/#/proteomeData (accessed on 10 July 2024)) to provide insights into how these mutations affect the likelihood that a protein folds properly. Unfolding fractions are also a continuous score, from 0 (the protein is stable and properly folded) to 1 (the protein is unstable and completely unfolded). Unfolding fractions were calculated by the UMS algorithm that our group previously developed [8,9].

Lastly, we extracted mutations with unknown clinical significance from ClinVar (https://www.ncbi.nlm.nih.gov/clinvar/ (accessed on 10 July 2024)) to use as a hold-out test set for our total of seven ML models. We analyzed 368 pathogenic mutations and 32,395 total amino acid variants across seven disease-related genes described for the first and second phases of the project, respectively. The list of the 368 pathogenic mutations is provided in the Supplementary Materials Table S1.

### 4.2. Determining the Relationship Between Protein Stability and Clinical Significance

We developed a program to compare average pathogenicity and average unfolding fractions—representing clinical significance and protein stability factors—quantitatively and qualitatively, at each amino acid residue in a protein. For example, TYRP1 contains 524 amino acid residues, each of which can potentially mutate to any of the other 19 amino acids. As a result, each residue in TYRP1 has 20 corresponding unfolding fractions (from UMS) and 20 pathogenicity scores (from EVE). Our program calculated the average unfolding fraction and pathogenicity scores for each residue and identified residues where both averages overlapped (i.e., unfolding fraction > 0.5 and pathogenicity score > 0.6). To calculate averages, we summed all pathogenicity scores and unfolding fractions, excluding the original amino acid, and then divided by 19. These values were then highlighted in red on the protein structure in the next-generation molecular visualization program from the Resource for Biocomputing, Visualization, and Informatics (RBVI), ChimeraX [52]. Highlighted residues were then compared to ClinVar mutations classified as “Pathogenic” or “Likely Pathogenic”.

We then used Decision Tree Regression to determine the R-squared value of the data. A Decision Tree Regression is a non-linear model that predicts outcomes by learning decision rules inferred from the data. The model works by recursively splitting the data based on learned decision rules to minimize the mean squared error across splits, then predicts the data, essentially trying to reproduce it. The R-squared value is calculated as the following:

$$R^{2}=1-\frac{{SS}_{res}}{{SS}_{tot}}$$

where *SS_res_* (Residual Sum of Squares) is the sum of the squares of the differences between the observed values and the values predicted by the model, and *SS_tot_* (Total Sum of Squares) is the sum of the squares of the differences between the observed values and the mean of the observed values. Of note, the mutations analyzed were single-missense mutations, which provides a limited view of how mutations can affect protein stability and function.

### 4.3. Data Preparation for Machine Learning Input

Of the 32,395 amino acid variants extracted for ML prediction, 716 had unknown clinical significance, according to ClinVar. These mutations are provided in the Supplementary Materials Table S2. Thus, our training and test set consisted of 31,679 and 716 protein variants, respectively. Our input consisted of the unfolding fractions for each amino acid in each protein sequence. For example, HBB has 147 amino acid residues, so 20 unfolding fractions for every possible single-missense mutation were input for each residue; hence, HBB made up 2940 (147 residues × 20 variants) of the 32,395 total amino acid variants extracted. Essentially, each protein mutant was identical to the wild-type variant, except for the mutated residue, which was substituted for its corresponding mutation. The ground-truth labels (pathogenic or benign) were derived from the pathogenicity score at the specific residue where the mutation occurred. If the score was greater than 0.6 or less than 0.4, we labeled the mutation as pathogenic or benign, respectively. All mutants with pathogenicity scores within this range (0.6 > x > 0.4) were not included in the original 32,395 variants extracted.

The length of the seven proteins varied, ranging from 147 to 586 amino acid residues. To ensure consistent input into the ML models, all inputs were standardized to a length of 586 residues; we padded variants with fewer than 586 residues. In addition, unfolding fractions were converted to scalars using the sci-kit-learn-1.5.2 Python module [56], to further pre-process the data before input into the ML algorithms. Scikit-learn is an open-source Python module with several algorithms used for ML and data analysis.

Due to a slight class imbalance in the training data, where there were more pathogenic than benign cases, all ML models were trained with class weights, ensuring that the models treated both classes (pathogenic or benign) equally during training and did not overfit to one class over another.

### 4.4. Comparison of Machine Learning Algorithms

A comparison of ML algorithms was evaluated on the hold-out test set to select the most effective prediction model. The four ML algorithms we considered were Decision Tree [57], Random Forest [57], Extreme Gradient Boosting (XGBoost) [58], and Support Vector Machine (SVM) [59]. The Decision Tree algorithm works by recursively splitting data based on features, creating a tree-like flowchart to make predictions. The Random Forest algorithm improves this by building multiple decision trees and merging their results to improve the accuracy of the prediction. XGBoost is a more advanced ML technique that builds a series of decision trees one after the other; each new tree tries to correct the mistakes made by the previous ones, leading to a very accurate overall prediction. Lastly, the SVM looks for the best way to separate the data into different categories to improve the accuracy of predictions.

Finally, the Decision Tree, Random Forest, and XGBoost algorithms were hypertuned with a max_depth of “None”, five minimum sample splits, one minimum sample leaf, and a random state of 42; the Random Forest and XGBoost algorithms were hypertuned with 100 and 400 estimators, respectively. The SVM algorithm was hypertuned with a linear kernel and a regularization of one.

### 4.5. Comparison of Neural Networks

We also evaluated the performance of three neural networks on the hold-out test set: a Dense model, an LSTM model, and an Ensemble network. The Dense model consisted of three fully connected layers of size 128, 64, and 32 nodes, respectively, with Rectified Linear Unit (ReLU) activation. ReLU activation is a function in ML that turns “on” when a signal is positive and stays “off” when it is negative, outputting a one or zero, respectively. Each fully connected layer was followed by a batch normalization and a 50% dropout layer. The final layer consisted of a fully connected layer of one node with sigmoid activation to produce a binary label, 1 (pathogenic) or 0 (benign). The LSTM model consisted of two LSTM layers with 64 nodes and tanh activation, separated by 50% dropout layers [21]. The final layer was the same as the Dense model’s. The Ensemble network combined both the Dense and LSTM models.

All networks were trained for 100 epochs with a batch size of 32, a validation split of 0.2, and the Adam optimizer with a learning rate of 1 × 10^−4^.

### 4.6. Performance Evaluation

We assessed the performance of all seven ML models to predict the pathogenicity of mutations with unknown clinical significance from ClinVar. Predictions were compared to pathogenicity scores from the EVE model. Each algorithm output was graded as true/false positive or true/false negative. From these values, accuracy, precision, recall, F1-score, AUC, and PR AUC scores were calculated. The equations for the calculation of the metrics are provided in Appendix A. Our criteria for a valid model prioritized AUC and PR AUC as the most important metrics, where an AUC score greater than 0.800 and a PR AUC score greater than 0.850 demonstrate a strong ability to distinguish between pathogenic and non-pathogenic mutations.

### 4.7. Structural Validation of Mutations with Unknown Clinical Significance

To validate our findings, we highlighted the predicted residues using ChimeraX-1.8, then subsequently analyzed the biochemical properties of the mutations, assessed their potential impact on protein structure and function, and compared the model’s predictions with the existing literature. Given the limited experimental data available for the predicted mutations, we also compared our predictions to additional computational findings from GeneDx [22] and Invitae (https://www.invitae.com/ (accessed 16 August 2024)) found on ClinVar, to further support our analysis.4.8. Statistical Analysis

To assess the significance of the results produced by each algorithm, we conducted a statistical analysis by employing bootstrapping methods. Specifically, we used *t*-tests on the bootstrapped samples to calculate *p*-values between the different models’ performances.

### 4.8. Code Availability

The code used for this paper is available at the following GitHub link: https://github.com/NIH-NEI/Predicting-Clinical-Significance-of-Single-Missense-Mutations-in-Ocular-Proteins (accessed on 10 July 2024). 

## 5. Conclusions

Our findings demonstrate the potential of protein stability as a predictive marker for assessing the clinical impact of single-missense mutations, particularly in ocular proteins. Among the machine learning models tested, the Random Forest algorithm emerged as the most robust, reinforcing its applicability in clinical genomics for predicting the pathogenicity of mutations with unknown significance. This study presents a novel framework that integrates protein stability data with machine learning approaches, advancing beyond traditional sequence-based or evolutionary models commonly used in the field. Compared to recent developments, our approach uniquely leverages structural insights to predict mutation impacts, aligning with current trends in precision medicine and computational biology. By incorporating protein stability parameters, we provide a more direct understanding of how genetic mutations influence protein function, particularly in key structural elements such as alpha-helices and beta-sheets. Furthermore, this approach holds promise for broader applications, including other disease-related proteins, and could be instrumental in guiding future therapeutic strategies. These may include the development of small molecules aimed at stabilizing native protein structures or targeted interventions that correct or compensate for the disrupted function caused by pathogenic mutations.

## Figures and Tables

**Table 1:** Overlap between high average-pathogenicity scores and average unfolding fractions in seven inherited disease-related ocular proteins.

Protein	Total Number of Residues	Number of Pathogenic Mutations	Number of Pathogenic-Unfolding Overlaps	Percent of Pathogenic-Unfolding Overlaps
DHDDS	333	11	9	72.70%
IMPDH1	514	13	9	69.20%
RPE65	533	99	95	96.20%
RHO	348	125	114	91.40%
KLHL7	586	12	11	94.40%
TYRP1	524	10	8	80.00%
HBB	157	66	60	75.80%

**Table 2:** Comparison of performances between the Decision Tree, Random Forest, XGBoost, and SVM ML algorithms.

Metrics	Decision Tree	Random Forest	XGBoost	SVM
AUC	0.896	0.922	0.882	0.762
F1-Score	0.813	0.811	0.721	0.604
Accuracy	0.852	0.849	0.782	0.690
Precision	0.759	0.725	0.680	0.569
Recall	0.875	0.878	0.768	0.643
PR AUC	0.859	0.879	0.816	0.657

**Table 3:** Comparison of performances between the Dense, LSTM, and Ensemble neural networks.

Metrics	Dense Model	LSTM Model	Ensemble Model
AUC	0.860	0.612	0.818
F1-Score	0.708	0.537	0.694
Accuracy	0.779	0.367	0.751
Precision	0.712	0.367	0.584
Recall	0.703	1.000	0.856
PR AUC	0.783	0.490	0.702

## Data Availability

The three datasets used were EVE pathogenicity scores (https://evemodel.org/ accessed on 10 July 2024), UMS unfolding fractions (https://neicommons.nei.nih.gov/#/proteomeData accessed on 10 July 2024), and clinical significance labels from ClinVar (https://www.ncbi.nlm.nih.gov/clinvar/ accessed on 10 July 2024).

## Supplementary Materials

The following supporting information can be downloaded at: https://www.mdpi.com/article/10.3390/ijms252111649/s1.

## Abbreviations

HBB: hemoglobin subunit beta; TYRP1: tyrosinase-related protein 1; RHO: rhodopsin; RPE65: retinal pigment epithelium-specific 65 kDa protein; DHDDS: dehydrodolichyl diphosphate synthase; IMPDH1: inosine-5′-monophosphate dehydrogenase 1; KLHL7: kelch-like protein 7; ML: machine learning; AUC: area under the curve; EVE: evolutionary model of variant effect; LSTM: long short-term memory network; PR AUC: precision–recall area under the curve; SVM: Support Vector Machine; UMS: unfolding mutation screen.

## Appendix A

The calculation of the performance metrics for this manuscript is as follows: precision (number of true positives/total number of true and false positives), recall (total number of true positives/total number of true positives and false negatives), F1-score (2 × precision × recall/[precision + recall]), and accuracy (number of true positives and true negatives/total number of entries). AUC and PR AUC were calculated automatically, using the scikit-learn-1.5.2 Python module [56].
