# Supplementary material for: Linking Protein Stability to Pathogenicity: Predicting Clinical Significance of Single-Missense Mutations in Ocular Proteins Using Machine Learning
Source: Int J Mol Sci. 2024 Oct 30;25(21):11649. doi: 10.3390/ijms252111649 (PMC11546782; doi:10.3390/ijms252111649)
Supplement: Supplementary file 1 [file ijms-25-11649-s001.zip › ijms-3279427-supplementary.pdf]

Article

Linking Protein Stability to Pathogenicity: Predicting Clinical Significance of Single-Missense Mutations in Ocular Proteins Using Machine Learning

Iyad Majid, Yuri V. Sergeev

Supplementary Materials

Supplementary Table S1. Relationship Between Pathogenic Mutations and Protein Stability Parameters Using Decision Tree Regression

For all seven proteins analyzed using Decision Tree Regression, a non-parametric algorithm that captures non-linear relationships, we observed an R-squared value of 0.846.

PROTEIN	Mutation	Average Pathogenicity	Average Unfolding Fractions
**HBB**	K83N	0.545	0.517
	L33Q	0.578	0.941
	H93Q	0.625	0.671
	H64N	0.632	0.447
	K83E	0.545	0.517
	V2L	0.604	0.915
	E27G	0.418	0.495
	E7M	0.372	0.881
	A54V	0.594	0.636
	E27D	0.418	0.495
	L107Q	0.552	0.811
	F43V	0.651	0.945
	H144Q	0.297	0.290
	G108D	0.512	0.695
	M1I	0.643	0.787
	Y36C	0.587	0.861
	R31G	0.566	0.591
	V24I	0.281	0.557
	H93N	0.625	0.671

V61E	0.608	0.867
H98L	0.549	0.639
S90R	0.635	0.945
A141T	0.609	0.968
D100A	0.620	0.944
V24F	0.281	0.557
A143D	0.378	0.781
V135E	0.311	0.750
F43L	0.651	0.945
D100N	0.620	0.944
D100G	0.620	0.944
D100V	0.620	0.944
D95H	0.358	0.405
K67T	0.548	0.516
R31S	0.566	0.591
L107R	0.552	0.811
H147P	0.575	0.395
K83T	0.545	0.517
K9E	0.486	0.607
H147D	0.575	0.395
F104L	0.632	0.926
P101L	0.623	0.951
Q128R	0.556	0.632
D100Y	0.620	0.944
D100H	0.620	0.944
H144P	0.297	0.290
L92P	0.460	0.820
E102D	0.426	0.509
K83M	0.545	0.517
H147L	0.575	0.395
E102K	0.426	0.509
N109K	0.277	0.373
Q128P	0.556	0.632
L29Q	0.609	0.885
C113R	0.106	0.308
F86S	0.480	0.958
L69H	0.513	0.938
K145N	0.242	0.674
E102G	0.426	0.509
A130P	0.544	0.930
A141V	0.609	0.968

	M1L	0.643	0.787
	S90N	0.635	0.945
	P125R	0.502	0.895
	E91D	0.384	0.608
	H98Q	0.549	0.639
	V110L	0.257	0.813
	Y146H	0.641	0.820
	L89P	0.638	0.800
	V35F	0.589	0.522
	L69P	0.513	0.938
	A116D	0.557	0.668
	V68E	0.612	0.409
	Y146N	0.641	0.820
	M1K	0.643	0.787
	H144R	0.297	0.290
	L33P	0.578	0.941
	L69F	0.513	0.938
	A28D	0.470	0.767
	F43S	0.651	0.945
	L29P	0.609	0.885
	M1V	0.643	0.787
	H93Y	0.625	0.671
	Y146C	0.641	0.820
	L115P	0.534	0.916
	V21M	0.369	0.291
	V127G	0.259	0.687
	V110M	0.257	0.813
	N20S	0.328	0.572
	R31K	0.566	0.591
	V99M	0.622	0.755
	M1T	0.643	0.787
	L111P	0.493	0.897
	M1R	0.643	0.787
	H64Y	0.632	0.447
	E122K	0.355	0.498
	A28S	0.470	0.767
	R31T	0.566	0.591
	E122Q	0.355	0.498
	E27K	0.418	0.495
	E7K	0.372	0.881
	E7V	0.372	0.881

**TYRP1**	I161F	0.675	0.920
	D86Y	0.702	0.860
	H224Y	0.840	0.280
	G433E	0.821	0.970
	F400L	0.899	0.540
	L382P	0.688	0.770
	C30R	0.897	0.970
	H215Y	0.862	0.990
	M1I	0.000	0.760
	R356Q	0.824	0.970
**RHO**	T193K	0.699	0.540
	V87F	0.466	0.920
	N15I	0.000	0.460
	G106V	0.828	0.950
	C110G	0.954	0.970
	P23T	0.000	0.950
	M163I	0.557	0.910
	E134K	0.772	0.500
	W161R	0.874	0.740
	L84P	0.706	0.940
	C187R	0.965	0.970
	G182E	0.784	0.970
	K296Q	0.961	0.800
	P23S	0.000	0.950
	Y191N	0.778	0.880
	L88P	0.424	0.830
	L131R	0.787	0.940
	A166V	0.461	0.620
	P180T	0.715	0.970
	T94I	0.563	0.860
	A169P	0.467	0.370
	C167W	0.626	0.820
	L46R	0.484	0.880
	S186W	0.759	0.780
	A292E	0.639	0.830
	L59H	0.797	0.830
	E113K	0.762	0.790
	N55K	0.948	0.750
	P23A	0.000	0.950
	D190E	0.758	0.860
	K296N	0.961	0.800

G284D	0.354	0.420
N78Y	0.877	0.340
P180R	0.715	0.970
G121V	0.816	0.950
G89R	0.414	0.950
L125R	0.762	0.930
P180S	0.715	0.970
C110W	0.954	0.970
G106A	0.828	0.950
N15K	0.000	0.460
V87D	0.466	0.920
P347A	0.000	0.620
S297R	0.728	0.740
G182V	0.784	0.970
G182D	0.784	0.970
P267T	0.975	0.890
P347S	0.000	0.620
V345A	0.000	0.490
M44T	0.680	0.940
H211P	0.619	0.830
P347T	0.000	0.620
G106R	0.828	0.950
H211R	0.619	0.830
G51V	0.757	0.950
G114V	0.848	0.970
N73S	0.786	0.430
V345L	0.000	0.490
M216R	0.363	0.870
T17K	0.000	0.210
V345E	0.000	0.490
S186P	0.759	0.780
R135P	0.892	0.830
M207R	0.618	0.950
M39R	0.224	0.820
P180A	0.715	0.970
S176F	0.802	0.940
L131P	0.787	0.940
R135G	0.892	0.830
P215A	0.911	0.950
P215L	0.911	0.950
P347Q	0.000	0.620

G114D	0.848	0.970
C185R	0.682	0.670
P180L	0.715	0.970
P171R	0.909	0.970
G109R	0.619	0.380
C187F	0.965	0.970
A164E	0.776	0.970
P23L	0.000	0.950
Q28H	0.000	0.890
C110Y	0.954	0.970
G182S	0.784	0.970
C187Y	0.965	0.970
D190G	0.758	0.860
Q28R	0.000	0.890
G51R	0.757	0.950
G90D	0.664	0.960
P347R	0.000	0.620
K296E	0.961	0.800
G188E	0.753	0.970
G106W	0.828	0.950
Y178H	0.880	0.880
R135L	0.892	0.830
C110F	0.954	0.970
P267L	0.975	0.890
P171L	0.909	0.970
V345M	0.000	0.490
E150K	0.189	0.760
P171S	0.909	0.970
M216K	0.363	0.870
D190N	0.758	0.860
P53R	0.680	0.770
P170R	0.793	0.960
N15S	0.000	0.460
G188R	0.753	0.970
T58R	0.770	0.660
C110R	0.954	0.970
D190Y	0.758	0.860
P171Q	0.909	0.970
Y178C	0.880	0.880
G89D	0.414	0.950
T17M	0.000	0.210

	A164V	0.776	0.970
	E181K	0.833	0.890
	P347L	0.000	0.620
	P23H	0.000	0.950
	R135W	0.892	0.830
**RPE65**	F326Y	0.488	0.840
	G241R	0.829	0.590
	D390N	0.000	0.920
	S382A	0.000	0.840
	A53D	0.686	0.970
	G436R	0.620	0.970
	L437F	0.439	0.920
	P271S	0.000	0.580
	H313P	0.848	0.950
	F70S	0.781	0.930
	P111T	0.719	0.940
	A415V	0.369	0.880
	E95Q	0.430	0.520
	G436V	0.620	0.970
	Y368C	0.620	0.930
	H144Q	0.682	0.930
	A145P	0.718	0.700
	H76P	0.205	0.740
	V172D	0.408	0.880
	G104R	0.706	0.960
	H241L	0.829	0.590
	V257G	0.421	0.900
	V136G	0.515	0.830
	P308L	0.402	0.880
	R305I	0.324	0.730
	G63R	0.755	0.920
	E335V	0.392	0.770
	F57S	0.538	0.930
	D186N	0.606	0.940
	F70V	0.781	0.930
	E148D	0.756	0.660
	G104V	0.706	0.960
	R91P	0.438	0.700
	H68Y	0.631	0.930
	F83L	0.598	0.950
	G528V	0.799	0.970

V19M	0.333	0.780
E381G	0.000	0.890
P467T	0.700	0.970
A179V	0.767	0.960
N135K	0.732	0.940
R118S	0.000	0.730
H182N	0.720	0.620
A415T	0.369	0.880
G104D	0.706	0.960
G48E	0.712	0.970
P467S	0.700	0.970
V287F	0.400	0.970
A360P	0.333	0.860
T306I	0.480	0.970
Y79H	0.728	0.930
M1I	0.000	0.750
R347C	0.500	0.930
R44P	0.752	0.770
F252S	0.665	0.950
F530L	0.748	0.940
P470L	0.753	0.850
A434E	0.487	0.780
I206T	0.642	0.920
V240F	0.380	0.800
H527R	0.864	0.820
I115T	0.000	0.700
P467A	0.700	0.970
Y249C	0.745	0.880
P111S	0.719	0.940
G140E	0.618	0.970
V473D	0.668	0.940
L67R	0.533	0.930
C330Y	0.335	0.760
G104S	0.706	0.960
M1T	0.000	0.750
G32C	0.814	0.970
D110G	0.695	0.750
G40D	0.857	0.970
Y144D	0.682	0.930
H182R	0.720	0.620
G484V	0.835	0.970

	R446S	0.348	0.750
	H182Y	0.720	0.620
	L408P	0.623	0.950
	H313R	0.848	0.950
	E417Q	0.776	0.830
	R81I	0.379	0.970
	V443A	0.320	0.850
	P363T	0.516	0.970
	Y318N	0.664	0.870
	Y239D	0.745	0.930
	D477G	0.473	0.300
	Y431C	0.670	0.940
	G187E	0.662	0.850
	R44Q	0.752	0.770
	L22P	0.295	0.940
	G484D	0.835	0.970
	P25L	0.592	0.870
	D167Y	0.573	0.960
	G40S	0.857	0.970
	L341S	0.000	0.850
	R515W	0.706	0.960
	Y368H	0.620	0.930
	R91W	0.438	0.700
	R91Q	0.438	0.700
**DHDDS**	R38H	0.830	0.566
	G54E	0.832	0.912
	S120N	0.451	0.870
	R37S	0.827	0.220
	P140R	0.125	0.494
	T113A	0.374	0.667
	R37C	0.827	0.220
	R205Q	0.875	0.732
	R211Q	0.832	0.633
	G35E	0.898	0.975
	K42E	0.415	0.689
**IMDPH1**	L160R	0.470	0.593
	L201R	0.859	0.960
	Q107P	0.183	0.962
	M86V	0.394	0.975
	S363P	0.669	0.919
	L251P	0.569	0.898

	K204N	0.774	0.661
	N193K	0.742	0.587
	Q318H	0.518	0.958
	N198K	0.494	0.256
	R224P	0.406	0.521
	D221G	0.352	0.699
	T310P	0.848	0.928
	K213R	0.506	0.694
	D226N	0.679	0.318
**KLHL7**	E451D	0.726	0.828
	L224P	0.265	0.949
	N145Y	0.796	0.823
	H375R	0.585	0.523
	W166C	0.283	0.546
	C110R	0.603	0.404
	A153T	0.767	0.919
	R372Q	0.701	0.523
	C421S	0.444	0.588
	R420C	0.751	0.749
	S150N	0.476	0.775
	A153V	0.767	0.919

Supplementary Table S2. Unfolding Fraction and Location of Mutations with Unknown Clinical Significance Predicted as Pathogenic

Locations of mutations with unknown clinical significance predicted as pathogenic by Random Forest for the seven proteins analyzed.

Protein	Mutation	Unfolding Fractions	Location
**HBB**	G47R	0.989	Loop
	N58K	0.646	Loop
**TYRP1**	Y248H	1.000	Loop
	C65Y	0.980	Sheet
	D212A	0.860	Loop
	G111R	0.960	Loop
	W90C	1.000	Loop
	A147D	1.000	Helix
	N96I	0.310	Loop

P247L	1.000	Loop
N280K	0.650	Loop
D123V	0.720	Loop
G217E	1.000	Loop
N132I	0.730	Loop
G61R	0.840	Loop
K152R	0.570	Helix
C99Y	1.000	Sheet
N186K	0.610	Helix
G57D	1.000	Helix
E413D	0.630	Helix
Q390H	0.540	Loop
M440K	1.000	Loop
Y464C	1.000	Sheet
D412N	0.410	Helix
F453S	0.990	Loop
S305I	0.980	Loop
N449D	0.990	Helix
H404Y	1.000	Active Site
S423C	0.820	Loop
G63S	1.000	Sheet
I479N	0.720	Loop
A456V	1.000	Helix
P328A	0.990	Loop
L514F	0.660	Loop
N435H	0.830	Loop
H224R	1.000	Active Site
G267E	1.000	Helix
I277M	0.990	Loop
I432F	1.000	Loop
F116Y	0.630	Loop

	F261L	0.930	Loop
	P346R	0.880	Loop
	I255S	0.890	Loop
	G361A	1.000	Loop
	G202R	0.990	Loop
	A235T	0.690	Helix
	L460V	0.950	Loop
	P470S	1.000	Loop
**RHO**	E181Q	0.790	Helix
	L172P	0.630	Helix
	W37S	1.000	Helix
	I179N	0.990	Helix
	S127Y	0.980	Helix
	F203S	1.000	Helix
	P171T	1.000	Helix
	I214N	0.910	Helix
	L59R	0.850	Helix
	S186L	0.970	Sheet
	R252H	0.720	Helix
	Y191D	1.000	Active Site
	R135Q	0.920	Helix
	L188P	0.990	Sheet
**RPE65**	G54V	1.000	Loop
	S242N	0.570	Active Site
	L38R	1.000	Loop
	H68P	1.000	Sheet
	P111H	0.990	Loop
	D186V	0.990	Loop
	I34T	0.970	Loop
	K294E	0.990	Loop
	S207C	0.476	Sheet

	A179T	0.990	Active Site
	G528R	1.000	Sheet
	G207C	0.426	Sheet
	R224G	0.904	Sheet
	R407W	0.674	Sheet
	G113V	0.987	Helix
	G380R	1.000	Loop
	R412G	0.705	Loop
	R341Q	0.747	Active Site
	D45G	0.895	Sheet
	K450E	0.817	Sheet
	S276F	0.421	Helix
	F114S	0.996	Loop
	S68F	1.000	Sheet
	R515Q	0.970	Sheet
	V85G	0.860	Loop
	L147R	0.491	Active Site
**DHDDS**	G35R	1.000	Loop
	Q277P	0.943	Helix
	L137S	0.997	Helix
	R222S	1.000	Helix
	Q184P	1.000	Loop
	G86C	1.000	Loop
	C306R	1.000	Helix
	R253C	0.857	Loop
	T306A	0.908	Helix
	L61P	1.000	Helix
	R205P	0.878	Sheet
	S275L	0.698	Helix
**IMPDH1**	G86R	1.000	Loop
	R407Q	0.822	Sheet

	Y208H	0.986	Sheet
	I91T	0.994	Loop
	K409E	0.982	Sheet
	A351E	0.994	Helix
	L252P	0.617	Loop
	P234L	0.764	Loop
	V336G	0.914	Helix
	L84V	0.993	Helix
	M467V	0.892	Helix
	R59C	0.947	Loop
	K238N	0.988	Loop
	R259C	0.780	Helix
	T393I	0.995	Helix
	R57W	0.837	Loop
	A236V	0.543	Loop
	T252S	0.905	Loop
	I332V	0.509	Helix
	R224C	0.685	Helix
	M325V	0.835	Loop
**KLHL7**	R407Q	0.822	Loop
	L117S	0.657	Helix
	Y124C	0.998	Helix
	Y385C	0.412	Sheet
	M77V	0.617	Loop
	R471T	0.895	Loop
	A163P	1.000	Loop
	V61M	0.206	Helix
	M262T	0.989	Helix
	G389S	1.000	Loop
	R222H	0.340	Helix
